# Validation of an Instrument to Measure Older Adults' Expectations Regarding Movement (ERM)

**DOI:** 10.1371/journal.pone.0043854

**Published:** 2012-08-24

**Authors:** Nabila Dahodwala, Jason Karlawish, Judy A. Shea, Cynthia Zubritsky, Matthew Stern, David S. Mandell

**Affiliations:** 1 Department of Neurology, University of Pennsylvania, Philadelphia, Pennsylvania, United States of America; 2 Department of Medicine, University of Pennsylvania, Philadelphia, Pennsylvania, United States of America; 3 Department of Psychiatry, University of Pennsylvania, Philadelphia, Pennsylvania, United States of America; University of Nebraska Medical Center, United States of America

## Abstract

**Background:**

Many individuals with Parkinson's disease are not diagnosed and treated. Attitudes about aging and related help-seeking may affect the timely diagnosis of Parkinson's disease. Our objectives were to develop measures of older adults' expectations regarding movement with aging, specifically related to parkinsonism, and their beliefs about seeking healthcare for the diagnosis and treatment of parkinsonism.

**Methods:**

We established content and face validity from interviews with experts, review of the literature, and pre-testing with key informants. Two 9-item instruments resulted: Expectations Regarding Movement (ERM) and Healthcare Seeking Beliefs for parkinsonism (HSB). These instruments were administered to 210 older adults at senior centers to investigate internal consistency and construct validity.

**Results:**

192 (91%) of the older adults completed more than 90% of the survey. The mean age was 76; 17 (9%) reported parkinsonism. Both scales demonstrated good internal consistency (α = 0.90). Factor analysis supported construct validity of the ERM and HSB scores. Older age, lower education, worse self-reported health and African American race each were associated with lower ERM scores, but not HSB scores.

**Conclusion:**

The ERM, a brief measure of expectations regarding movement with aging, shows reliability and validity. This scale may be useful in identifying older adults at increased risk for under-identification of Parkinson's disease. Further work is needed to measure healthcare seeking for parkinsonism.

## Introduction

Parkinson's disease (PD) is prevalent and has high social, economic and psychological costs. A diagnosis of PD relies upon two elements: a patient's symptom report and a physician's neurological history and exam. Early and accurate diagnosis is important because treatment leads to both increased survival [1] and improved clinical outcomes [2], [3], [4], [5], [6], [7].

Unfortunately, there is a gap between prevalent PD cases and the smaller number of people who are diagnosed and receive treatment. Epidemiological studies have shown that between 12%–78% of PD cases are undiagnosed [8], [9], [10], [11], [12], [13], [14]. Furthermore, African-Americans with PD are twice as likely than whites to be undiagnosed [13]. Researchers propose several reasons for the large diagnostic gap in PD. These include the insidious onset of symptoms, lack of appropriate access to primary or specialty care, and misinterpretation of symptoms as normal aging [15]. One small study of 74 older adults with PD suggested that individuals who under-reported disability symptoms had delayed diagnosis of PD [16].

A better understanding of why many older adults with PD do not get diagnosed and treated or have delayed diagnoses is critical to the design of interventions to reduce the gap between prevalent cases and diagnosed patients. Since one of the initial steps in a path to PD diagnosis is the older adult considering movement-related symptoms to be abnormal, it is important to understand expectations regarding movement with aging and associated healthcare seeking beliefs. If barriers to appropriate diagnosis are due, at least in part, to these patient expectations, then improving healthcare access or medical education will not succeed as well as expected. Instead, interventions to improve the timely diagnosis of PD would need to target older adults' expectations of age-related movement. Factors that could mediate the relationship between aging beliefs and healthcare seeking for PD include demographics, socioeconomic status, medical and cultural beliefs which include the concepts of religiosity and collectivism. Cultural beliefs, in particular, have been shown to give meaning to aging-related illnesses such as Alzheimer's disease [17] and could influence aging expectations.

A growing body of literature suggests that older adults frequently attribute health problems to normal aging [18], [19], [20], [21]. When older adults attribute health problems to normal aging they will not believe it important to seek healthcare [22]. This, in turn, may lead to delays in treatment [23] and increased mortality [24]. Studies of this relationship between aging expectations and healthcare seeking behaviors to date, however, have focused on cognition and mood. They have not addressed movement, a critically important domain associated with neurodegenerative diseases of aging, especially PD.

The purpose of this study, therefore, was to develop instruments to measure expectations of movement with aging and beliefs about seeking healthcare for parkinsonism. Our goal was to generate items, refine the content and test the psychometric properties of the instruments. We hypothesized that worse scores on the instruments will be associated with greater age, less education and poorer self-reported health.

## Methods

### Instrument Development

To construct the Expectations Regarding Movement (ERM) instrument, we consulted experts in the fields of internal medicine, geriatrics and neurology through two one-hour conferences to compile a battery of instruments to measure expectations and beliefs about aging. Next, we reviewed these instruments, including their bibliographies, to find any additional instruments to measure expectations and beliefs. We identified the Expectations Regarding Aging-12 (ERA-12) [25], as the scale that most closely related to our construct of expectations about movement. The ERA-12 assesses expectations about general physical health, cognitive function and mental health with aging. After consulting with the author of the instrument, we adapted the ERA-12 to address the domain of movement-related function. The symptoms of parkinsonism included in this domain were drawn from the 9-item “Brief Screening Questionnaire for Parkinsonism [26].” The 9 items designed to measure the ERM included the stem and response options from the ERA-12 combined with PD symptoms from the parkinsonism screening questionnaire [**Table S1**]. For example, subjects were asked to respond on a 4-point Likert scale to statements such as, “Shuffling your feet and taking tiny steps when walking is just something that happens when you get old.”

We used a similar approach to develop the second instrument, Healthcare Seeking Beliefs for parkinsonism (HSB). This 9-item scale utilized the same question style and wording as a scale that measures healthcare seeking beliefs in general [27], but replaced the content with symptoms of PD. Lower scores on the original healthcare seeking beliefs scale were associated with older age and lower aging expectations [27]. Subjects were asked to respond on a 3-point scale of importance whether parkinsonian symptoms should be discussed with a doctor. For example, “If an older friend's arms or legs shake, how important is this to discuss with a doctor?”

Draft items were tested on 17 key informants through two iterations. This group consisted of consumers of health care and mental health professionals who have worked on the development of scales for national surveys. They were asked to complete a survey response form that included space for comments and the overall comprehensibility, level of interest and intrusiveness of the instruments. The introduction to each instrument, formatting, individual response items and question wording were modified based on these results.

We calculated readability by using the following formula (Flesch-Kincaid reading level): (.39×ASL)+(11.8×ASW)–15.59, where: ASL = average sentence length (the number of words divided by the number of sentences) and ASW = average number of syllables per word (the number of syllables divided by the number of words). The ERM and HSB scales each had a Flesch-Kincaid reading level of grade 6.4.

### Participants

We collected data from 210 community-residing older adults attending all eight Delaware County, Pennsylvania senior centers. The study investigators went to each center to explain the study's purpose: to better understand older adults' expectations and beliefs about aging, and to invite English speaking adults over the age of 55 to participate in the study. Participants were blinded to the study hypotheses and completed the survey anonymously in a semi-supervised setting.

### Ethics Statement

The University of Pennsylvania Institutional Review board approved the study protocol with a waiver of documentation of informed consent. All participants provided verbal informed consent prior to entry into the study. We did not obtain written informed consent given the study's minimal risk, and greater potential risk involved with obtaining written signatures for this anonymous study.

### Covariates

To study the relationships between the ERM and HSB, we gathered the following information: 1) 5 demographic items (age, sex, ethnicity, self-reported race, education); 2) a screening questionnaire for parkinsonism [26], and; 3) an item from the Medical Outcomes Study Short Form-12 to measure self-reported health. This item asked, “In general, would you say your health is: Excellent, Very Good, Good, Fair or Poor?” We also administered the following instruments that capture similar concepts to aging expectations and beliefs to assess the construct validity of the ERM and HSB: 1) Expectations Regarding Aging-12 (ERA-12) [25], to measure general expectations about aging; and 2) cultural scales to measure collectivism and religiosity [28].

### Analyses

#### Descriptive statistics

We examined the frequencies of responses to each item and assessed ceiling and floor effects. Mean scores for each instrument were transformed linearly to a 0–100 possible range, to provide easier to interpret comparisons across scales with different item numbers and response options. Lower scores on the ERM indicate that the respondent expects the development of parkinsonism to be a part of normal aging. In contrast, higher scores indicate that the respondent expects to age successfully with regard to movement. Similarly, lower scores on the healthcare seeking beliefs for parkinsonism (HSB) indicate that the respondent believes it was not important to discuss parkinsonian symptoms with a doctor.

For missing items, we imputed a value based on the average item score for that scale across all participants. Individuals with less than 90% of the total survey complete or >50% of any individual scale missing were dropped from the analysis. We calculated the mean, median, standard deviation and range of scores for each instrument.

#### Reliability

We used Cronbach's coefficient alpha to measure the ERM and HSB's internal consistency and interpreted a coefficient between 0.70 and 0.90 as indicating adequate scale reliability.

#### Content Validity

To assess content validity we reviewed the literature, consulted experts and administered the survey to key informants and reviewed their responses to survey.

#### Construct Validity

To test construct validity, we performed exploratory factor analysis with principal components extraction. The goal was to observe if the five scales included in our survey measures – the ERM, HSB, ERA-12, collectivism and religiosity - represented separate, but related constructs. We expected to observe separate and distinct factors for each of the five scales. Moreover, because we expected the components to be related we opted for oblique (correlated) rotation; specifically we used the PROMAX solution. We used the common practices of retaining the number of factors equal to the number of eigenvalues >1, balanced with an examination of the scree plot. Based on these criteria 7 factors were included for the final interpretation. Items with rotated loadings (or correlation coefficients between the item and the factor) above 0.50 were interpreted as correlating well with the corresponding component.

We also performed extreme group comparisons between age, education, and self-reported health with the ERM and HSB using two sided *t*-tests [29]. We hypothesized that older age, less education and poorer health would be associated with lower scores on both the ERM and HSB.

## Results

### Sample characteristics

One hundred and ninety-two of the 210 participants (91%) completed at least 90% of the questionnaire items. The mean respondent age was 76 years (**Table 1**). Most were women (78%). The racial/ethnic mix of the sample was 62% white, 31% African-American and 2% Latino. Almost half (41%) had completed more than high school. The majority of respondents (83%) described their health as excellent, very good or good.

**Table 1:** Sample characteristics.

*Demographic and clinical*		*N = 192*	*%*
Mean age in years (SD)		75.7 (8.8)	
Female		150	78
Race/ethnicity^a^	White	119	62
	African-American	60	31
	Latino	3	2
	Other	8	5
Education^b^	Less than high school	33	18
	High school	79	41
	More than high school	77	41
Parkinsonism	Diagnosis of PD	5	3
	Positive screen for symptoms	17	9
Self-reported health^a^	Excellent, very good or good	160	83
	Fair or poor	30	16

a 2 subjects with missing items

b 3 subjects with missing items

Two participants did not complete any portion of the HSB scale. This scale also had 18 respondents (9%) who left between 1 and 3 items blank. Nine (5%) respondents had 1 item missing and 1 respondent had 4 items missing on the ERM scale. The overall frequency of missing items for both scales was 1%. There were no significant differences in baseline characteristics between participants who were excluded due to insufficient information, and those who remained in the analysis.

### Reliability Analysis

**Table 2** shows the descriptive statistics and internal consistency estimates for all scales. Both the ERM and HSB had a Cronbach's alpha = 0.90.

**Table 2:** Descriptive statistics and reliability.

*Scale*	*# of items*	*Mean score*	*Median score*	*SD*	*Observed Range (Possible range)*	*Internal consistency*
ERM^a^	9	57	59	17.5	0–100 (0–100)	0.90
HSB^a^	9	54	50	28.0	0–100 (0–100)	0.90
ERA-12^a^	12	50	50	15.9	0–86 (0–100)	0.87
Collectivism^a^	6	80	86	19.8	0–100 (0–100)	0.80
Religiosity^a^	9	70	74	24.5	0–100 (0–100)	0.93
Parkinsonism screen	9	12	6	16.3	0–67 (0–100)	0.82

a Scores were scaled from 0 to 100

### Factor Analysis

An exploratory principal components analysis revealed nine components with eigenvalues greater than 1. Seven factors that explain 62% of the total variance were interpreted. The rotated factor loadings for each of the 45 items within the 5 scales are provided in **Table S2**. In this table we highlight the rotated loadings >0.50. Rotated loading are in yellow when they are within a scale as expected and have a loading >.50. The two items loading within the expected factor (scale) with loadings <.50 are in blue. Four items highlighted in green when the factor loading exceeded .50 or has its highest loading (regardless of magnitude) on an alternative/unexpected factor.

Items for our new scale of Expectations Regarding Movement (ERM) instrument are in the third component. Here factor loadings ranged from 0.58–0.84. Note however, that two items also had sizable loading with an Expectations Regarding Aging component. Items for Healthcare Seeking Beliefs for parkinsonism (HSB) are in the second component, with loadings ranging from .64–.83.

Two of the established scales from prior work stood alone as unique constructs. The constructs of religiosity and collectivism were captured in the first and fourth components, respectively, with rotated factor loadings ranging from 0.54–0.92, with the exception of the first item in the collectivism scale. The last three components were comprised primarily of items developed to measure expectations regarding aging in general: cognitive health, mental health and physical health expectations, although for the latter two items loaded best on alternative components.

### Between-group comparisons

As hypothesized, the comparison of ERM scores between extreme groups revealed that on average, subjects who were younger (<70), had attained more formal education (>high school) or had better self-reported health (very good or excellent) were more likely to report higher expectations for movement than subjects who were older (>82), had attained less formal education (<high school) and had fair or poor self-reported health (**Table 3**). In addition to extreme groups, African Americans and whites differed significantly in the ERM scores but not their ERA scores. While African Americans had lower expectations regarding movement (mean score 53.1 for African Americans, 59.6 for whites, *p* = 0.01), there was no difference in expectations about aging in general (mean score 51.0 for African Americans, 50.0 for whites; *p* = 0.68).

**Table 3:** Comparison of Expectations Regarding Movement (ERM) survey scores and Health Seeking Beliefs (HSB) for parkinsonism survey scores between groups.

*Construct*	*Subcategory*	*Mean ERM score*	*p-value*	*Mean HSB score*	*p-value*
Age	1^st^ quartile (age<70)	60.3	0.04	60.9	0.39
	4^th^ quartile (age>82)	52.3		55.9	
Education	Less than high school	45.6	<0.0001	56.0	0.62
	More than high school	64.2		58.9	
Self-reported health	Poor or fair	49.3	0.003	57.6	0.61
	Very good or excellent	61.8		54.3	
Race	African American	53.1	0.01	54.6	0.60
	White	59.6		52.4	

Scores on the HSB instrument were not significantly associated with any of the expected clinical or demographic characteristics.

## Discussion

This study provides evidence for the reliability and validity of the ERM, a 9-item instrument that measures expectations among older adults regarding movement. It provides weaker evidence for the scale measuring healthcare seeking beliefs for parkinsonism, which had excellent internal consistency but did not meet our standards for construct validity. The use of the ERM instrument in subsequent research studies may help to explain the role of older adults' expectations in contributing to the diagnostic gap in PD, particularly among African-Americans.

Among our sample of community-dwelling older adults, the ERM scale demonstrated excellent internal consistency and good validity. Factor analysis showed that the constructs of Expectations Regarding Aging and Expectations Regarding Movement were closely related to each other, but measured different constructs. While the ERM measures expectations regarding the development of parkinsonism with aging, the ERA measures constructs related to cognitive, mental and general physical health (e.g. energy, pain).

The construct of movement-related expectations was further validated when scores on the ERM scale were compared by race. The finding that African Americans were more likely than whites to expect to develop parkinsonism informs prior findings regarding the relationship between race and PD. African Americans are less likely to receive a diagnosis of PD than whites [30], [31], [32], [33], [34], [35]. This suggests that the lower rates of PD diagnosis in African Americans may be related to lower expectations about movement.

Further validating the construct of expectations about movement were the associations seen with age, education and health status. Old age has previously been shown to correlate with attributing symptoms of disability to age itself [18]. Educational attainment likely relates to health knowledge which in turn affects expectations. Lastly, those individuals with worse health status were more likely to have lower expectations. This finding corresponds with earlier reports that the standard by which individuals rate health is dependent on their own health [36].

This study provided some evidence for the construct validity of the instrument developed to measure healthcare seeking beliefs for parkinsonism (HSB). It is possible that this 9-item instrument only captured a portion of what motivates health behavior. Published health belief models include expectations about aging or perceived severity as one factor that influences health behavior [37]; there are many other factors in the health belief model such as perceived susceptibility, perceived benefits (knowledge of treatment availability), perceived barriers (material and psychological costs), cues to action and self-efficacy that are important to measure to gain a complete understanding of what motivates healthcare seeking. Subsequent measures of healthcare seeking beliefs for parkinsonism should incorporate these other domains to adequately assess this construct.

This study had the following limitations. First, there is no gold standard test to measure expectations about movement. Therefore, we could not test criterion validity. Second, we also were not able to assess for test-retest reliability given the constraints of anonymous testing. Future longitudinal studies can assess both test-retest reliability and correlate ERM scores with health service use and health outcomes to further support score validity. Third, the use of a convenience sample may have introduced some sampling bias in the study responses. The predominantly women sample who attend senior centers may represent more active and health-conscious members of the community. This will limit the generalizability of the results.

Despite these limitations, this study is an important first step in developing an instrument that measures expectations about movement. Information collected through measures of aging expectations in general has been instrumental in developing targeted interventions to improve health in other areas of aging. Studies have shown that older adults with low expectations about physical activity and depression are less likely to seek healthcare [22], [38]. These findings have led to interventions to improve expectations and, in turn, health outcomes [39].

Based on our results, measuring expectations about movement may be an important first step in identifying individuals at-risk for the under-identification of PD. After identifying sub-groups of older adults that are at highest risk for low expectations and subsequent healthcare seeking, targeted interventions based on improving expectations can be implemented.

## Supporting Information

Table S1 9-item Expectations Regarding Movement (ERM) questionnaire.
(DOC)

Table S2 **Exploratory factor analysis, rotated factor structure (oblique): an analysis of the correlation of each questionnaire item with the constructs of aging expectations, religiosity, and collectivism.** ^a^ERM = Expectations Regarding Movement; ^b^ERA = Expectations Regarding Aging; ^c^HSB = Healthcare Seeking Beliefs for parkinsonism; ^d^COL = Collectivism scale; ^e^REL = Religiosity scale. Factor loadings are highlighted in yellow when they are within the expected scale and have a loading >0.50. Factor loadings are highlighted in green when they have a loading >0.50, but are within an unexpected/alternative factor OR when the highest loading, regardless of magnitude, is within an unexpected/alternative factor. Factor loadings are highlighted in blue when they are within the expected scale, but have a loading <0.50.
(DOC)
