# Supplementary material for: Validation of an Instrument to Measure Older Adults' Expectations Regarding Movement (ERM)
Source: PLoS One. 2012 Aug 24;7(8):e43854. doi: 10.1371/journal.pone.0043854 (PMC3427187; doi:10.1371/journal.pone.0043854)
Supplement: Table S1 — 9-item Expectations Regarding Movement (ERM) questionnaire. (DOC) [file pone.0043854.s001.doc]

Supporting text

9-item Expectations Regarding Movement (ERM) questionnaire

1. Having problems getting out of a chair is an accepted part of aging.
2. When people get older, their handwriting will become smaller.
3. Every year that people age, their voice will become softer.
4. Problems with balance, when walking or standing, are a natural occurrence just from growing old
5. When people get older, they will suddenly seem to get stuck in doorways.
6. As people get older, their face will become less expressive than it used to be.
7. It’s normal for your arms or legs to shake when you are old.
8. It’s an accepted part of aging to have trouble buttoning buttons.
9. Shuffling your feet and taking tiny steps when walking is just something that happens when you get old.
