# Supplementary material for: Validation of an Instrument to Measure Older Adults' Expectations Regarding Movement (ERM)
Source: PLoS One. 2012 Aug 24;7(8):e43854. doi: 10.1371/journal.pone.0043854 (PMC3427187; doi:10.1371/journal.pone.0043854)
Supplement: Table S2 — Exploratory factor analysis, rotated factor structure (oblique): an analysis of the correlation of each questionnaire item with the constructs of aging expectations, religiosity, and collectivism. aERM = Expectations Regarding Movement; bERA = Expectations Regarding Aging; cHSB = Healthcare Seeking Beliefs for parkinsonism; dCOL = Collectivism scale; eREL = Religiosity scale. Factor loadings are highlighted in yellow when they are within the expected scale and have a loading >0.50. Factor loadings are highlighted in green when they have a loading >0.50, but are within an unexpected/alternative factor OR when the highest loading, regardless of magnitude, is within an unexpected/alternative factor. Factor loadings are highlighted in blue when they are within the expected scale, but have a loading <0.50. (DOC) [file pone.0043854.s002.doc]

Table S2. Exploratory factor analysis, rotated factor structure (oblique): an analysis of the correlation of each questionnaire item with the constructs of aging expectations, religiosity, and collectivism

|  | Factor #1: Religiosity | Factor #2: Health seeking beliefs | Factor #3: Movement expectations | Factor #4: Collectivism | Factor #5: Cognitive health expectations | Factor #6: Mental health expectations | Factor #7: Physical Health expectations |
| --- | --- | --- | --- | --- | --- | --- | --- |
| Eigenvalue | 8.98 | 6.32 | 5.31 | 2.66 | 1.90 | 1.40 | 1.27 |
| % of Variance | 20 | 14 | 12 | 6 | 4 | 3 | 3 |
| ERM^a^ getting out of chair | -0.06 | 0.13 | **0.58** | 0.18 | 0.65 | 0.32 | 0.11 |
| ERM^a^ change handwriting | -0.06 | 0.15 | **0.76** | -0.02 | 0.32 | 0.32 | 0.19 |
| ERM^a^ voice softer | -0.05 | 0.10 | **0.66** | 0.13 | 0.54 | 0.48 | 0.12 |
| ERM^a^ balance | -0.11 | 0.11 | **0.68** | 0.01 | 0.49 | 0.30 | 0.09 |
| ERM^a^ sudden freezing | -0.07 | 0.04 | **0.81** | 0.12 | 0.33 | 0.34 | 0.28 |
| ERM^a^ face less expressive | -0.13 | 0.13 | **0.77** | 0.19 | 0.32 | 0.37 | 0.20 |
| ERM^a^ shaking of limbs | -0.11 | 0.07 | **0.84** | 0.18 | 0.24 | 0.37 | 0.35 |
| ERM^a^ difficulty buttoning | -0.22 | 0.15 | **0.67** | 0.14 | 0.41 | 0.25 | 0.43 |
| ERM^a^ shuffling gait | -0.17 | 0.12 | **0.73** | 0.17 | 0.40 | 0.49 | 0.19 |
| ERA^b^ health | -0.04 | 0.10 | 0.43 | 0.06 | 0.22 | 0.52 | **0.54** |
| ERA^b^ aches | -0.05 | 0.09 | 0.42 | 0.09 | 0.31 | 0.35 | 0.35 |
| ERA^b^ worn out | -0.05 | -0.04 | 0.34 | 0.07 | 0.38 | 0.35 | **0.78** |
| ERA^b^ energy | -0.14 | -0.01 | 0.41 | -0.01 | 0.61 | 0.30 | **0.60** |
| ERA^b^ family time | -0.01 | 0.03 | 0.42 | 0.11 | 0.22 | **0.74** | 0.32 |
| ERA^b^ lonely | -0.09 | -0.04 | 0.42 | 0.12 | 0.43 | **0.79** | 0.17 |
| ERA^b^ worry | -0.12 | 0.02 | 0.37 | 0.03 | 0.48 | **0.67** | 0.33 |
| ERA^b^ depressed | -0.07 | -0.01 | 0.44 | 0.18 | 0.45 | **0.67** | 0.42 |
| ERA^b^ forgetful | -0.05 | -0.04 | 0.25 | 0.02 | **0.67** | 0.47 | 0.29 |
| ERA^b^ names | -0.16 | 0.07 | 0.33 | 0.10 | **0.72** | 0.27 | 0.17 |
| ERA^b^ natural to forget | -0.09 | 0.03 | 0.41 | -0.08 | **0.70** | 0.33 | 0.10 |
| ERA^b^ mental slowing | -0.11 | 0.07 | 0.42 | -0.01 | **0.71** | 0.22 | 0.30 |
| HSB^c^ getting out of chair | 0.17 | **0.73** | 0.03 | 0.27 | 0.04 | 0.01 | -0.08 |
| HSB^c^ change handwriting | -0.01 | **0.70** | -0.04 | 0.00 | 0.17 | -0.19 | -0.23 |
| HSB^c^ voice softer | -0.05 | **0.64** | -0.07 | 0.05 | 0.14 | 0.33 | -0.09 |
| HSB^c^ balance | -0.01 | **0.75** | 0.14 | 0.30 | 0.02 | 0.07 | 0.03 |
| HSB^c^ sudden freezing | 0.04 | **0.79** | 0.17 | 0.20 | 0.03 | -0.02 | -0.05 |
| HSB^c^ face less expressive | -0.09 | **0.76** | 0.06 | 0.05 | 0.15 | -0.12 | -0.10 |
| HSB^c^ shaking of limbs | 0.03 | **0.78** | 0.25 | 0.40 | 0.05 | 0.08 | 0.16 |
| HSB^c^ difficulty buttoning | 0.01 | **0.83** | 0.11 | 0.23 | 0.04 | -0.04 | 0.03 |
| HSB^c^ shuffling gait | -0.08 | **0.81** | 0.21 | 0.34 | 0.09 | 0.02 | 0.20 |
| COL^d^ relatives stay | 0.12 | 0.28 | -0.14 | 0.35 | -0.07 | -0.13 | -0.03 |
| COL^d^ turn to each other | 0.06 | 0.33 | 0.21 | **0.76** | 0.05 | 0.09 | 0.02 |
| COL^d^ raise children | -0.04 | 0.21 | 0.12 | **0.62** | 0.16 | -0.08 | 0.35 |
| COL^d^ care for older family | -0.06 | 0.25 | 0.14 | **0.77** | 0.12 | -0.01 | 0.23 |
| COL^d^ communicate often | 0.10 | 0.23 | 0.05 | **0.76** | -0.13 | 0.16 | -0.04 |
| COL^d^ help move ahead | 0.20 | 0.17 | 0.10 | **0.82** | -0.04 | 0.15 | 0.01 |
| REL^e^ talk about faith | **0.54** | -0.04 | 0.00 | -0.01 | -0.23 | 0.10 | -0.02 |
| REL^e^ read religious books | **0.78** | -0.04 | -0.10 | -0.01 | -0.10 | 0.05 | -0.08 |
| REL^e^ watch religious show | **0.80** | -0.02 | -0.20 | -0.06 | -0.13 | 0.10 | -0.08 |
| REL^e^ beliefs are foundation | **0.80** | 0.05 | -0.04 | 0.11 | -0.08 | -0.05 | -0.04 |
| REL^e^ presence of god | **0.87** | 0.02 | -0.04 | 0.12 | -0.14 | -0.01 | 0.02 |
| REL^e^ relationship with god | **0.90** | 0.06 | -0.11 | 0.12 | -0.14 | -0.03 | -0.10 |
| REL^e^ pray for healing | **0.87** | -0.01 | -0.06 | 0.11 | -0.22 | 0.04 | -0.11 |
| REL^e^ pray often | **0.92** | -0.01 | -0.07 | 0.06 | -0.18 | -0.01 | -0.10 |
| REL^e^ rely on god for health | **0.83** | 0.01 | -0.24 | -0.04 | -0.15 | -0.13 | -0.14 |

^a^ERM = Expectations Regarding Movement; ^b^ERA = Expectations Regarding Aging; ^c^HSB = Healthcare Seeking Beliefs for parkinsonism; ^d^COL = Collectivism scale; ^e^REL = Religiosity scale

Factor loadings are highlighted in yellow when they are within the expected scale and have a loading >0.50

Factor loadings are highlighted in green when they have a loading >0.50, but are within an unexpected/alternative factor OR when the highest loading, regardless of magnitude, is within an unexpected/alternative factor.

Factor loadings are highlighted in blue when they are within the expected scale, but have a loading <0.50
